# Rapid risk assessment from ECDC: Resurgence of reported cases of COVID-19 in the EU/EEA, the UK and EU candidate and potential candidate countries

**DOI:** 10.2807/1560-7917.ES.2020.25.26.2007021

**Published:** 2020-07-02

**Authors:** Eurosurveillance editorial team

**Affiliations:** 1 European Centre for Disease Prevention and Control (ECDC), Stockholm, Sweden

The European Centre for Disease Prevention and Control (ECDC) provides regularly updated information on coronavirus disease-2019 (COVID-19) relevant to Europe on a dedicated webpage. Besides general information including Q&As, daily case counts, and maps with disease distribution, examples of latest updates comprise: COVID-19 Aviation Health Safety Protocol: Guidance for the management of airline passengers in relation to the COVID-19 pandemic, Heating, ventilation and air-conditioning systems in the context of COVID-19, and Guidance on infection prevention and control of COVID-19 in migrant and refugee reception and detention centres in the EU/EEA and the UK. ECDC also publishes regular risk assessments and the Box below contains the summary from the rapid risk assessment published on 2 July 2020.

Box **Summary of the ECDC rapid risk assessment from 2 July 2020**

On Since 31 December 2019 and as of 30 June 2020, 10,273,001 cases of coronavirus disease-2019 (COVID-19) have been reported worldwide, including 505,295 deaths. EU/EEA countries and the UK have reported 1,556,709 cases (15% of all cases), including 176,800 deaths (35% of all deaths), while EU candidate and potential candidate countries reported 229,112 cases (2% of all cases), including 5,988 deaths (1% of all deaths).

The COVID-19 pandemic is posing an unprecedented threat to EU/EEA countries and the UK as well as countries worldwide, many of which have been experiencing widespread transmission of the virus in the community for several months. While decreasing trends in disease incidence are being observed in Europe overall (12% decrease in 14-day incidence of reported cases between 16 and 30 June), there is still community transmission reported in most EU/EEA countries, the UK and EU candidate and potential candidate countries. Additionally, some countries are reporting a resurgence of observed cases or large localised outbreaks.

The reasons behind this apparent increase in the number or resurgence of cases observed in these countries vary. The increase in the number of cases may reflect changes in case ascertainment (e.g. increasing testing, changes in the case definition) that does not necessarily indicate increased rates of transmission, or may reflect genuine increases in transmission (e.g. associated with the easing of non-pharmaceutical interventions (NPI), large localised outbreaks), or may be due to importation of cases. Some of the observed increases, particularly in countries with a small population, are associated with just a few additional new cases.

Therefore, information must be interpreted with caution.

In this risk assessment, ECDC is assessing the risks associated with these reported increases of incident cases in some countries.

Currently, the risk, determined by a combination of the probability of an event occurring and of its consequences (impact) to individuals or the population, is assessed as follows:

- The overall risk of COVID-19 in countries reporting an increase in incident COVID-19 cases and for which there is, or may shortly be, substantial ongoing community transmission and/or within which appropriate physical distancing measures are not taken, is currently considered **moderate** for the general population (very high probability of infection and low impact of disease) and **very high** for populations with defined factors associated with elevated risk for COVID-19 (very high probability of infection and very high impact of disease).
- Provided that the increases are not merely reflecting a change in the surveillance strategy or artefacts due to small number calculations; the overall risk of COVID-19 transmission further rising in these countries with observed increase of COVID-19 incidence is considered **high** (very high probability of further increase and moderate impact of a further increase) if no appropriate monitoring systems and capacities for extensive testing and contact tracing are in place, and if NPIs are eased when there is still ongoing community transmission.

In order to respond to these risks, the following measures continue to be essential to maintain a reduced level of transmission and avoid resurgence:

- A robust monitoring framework to closely monitor the epidemiological situation, rapidly detect increased transmission, assess the impact of the interventions in place and avoid a resurgence of COVID-19.
- An expanded testing strategy aimed at comprehensive testing of all individuals displaying symptoms compatible with COVID-19, independent from their country of origin or residency.
- A framework for contact tracing, based on extensive testing, active case finding, early detection of cases, isolation of cases, quarantine and follow-up of contacts, possibly supported by electronic tools and applications.
- Prompt identification and investigation of clusters/outbreaks associated with specific settings, with implementation of tailored control and prevention measures to minimise onward spread to others in the setting and to the wider community.
- Long-term sustainable implementation of essential NPIs, irrespective of transmission rates, and the ability to amend strategies rapidly in response to indications of increased transmission, if appropriate, only restricting those to subnational areas.
- A strong risk communication strategy should remind citizens that the pandemic is not over.

National authorities should consider carefully analysing every increase in incidence to assess whether these are associated with genuine increases in transmission and whether these involve populations with defined factors associated with elevated risk for COVID-19, including the residents of long-term care facilities (LTCFs). Identifying possible outbreaks, other foci of transmission, or sustained community transmission due to the easing of the NPIs imposed in previous months is essential to control such increases in incidence and implement tailored control measures aimed at limiting population mobility and/or reducing exposure.

ECDC does not consider travel restrictions within and to the Schengen area as an efficient way to reduce transmission within the EU since community transmission is already taking place in the EU/EEA and data from The European Surveillance System (TESSy) show that, in June 2020, only 3% of confirmed cases were likely infected in a country different from the reporting country.

Source: European Centre for Disease Prevention and Control (ECDC). Resurgence of reported cases of COVID-19 in the EU/EEA, the UK and EU candidate and potential candidate countries. Stockholm: ECDC; 2 July 2020. Available from: https://www.ecdc.europa.eu/sites/default/files/documents/RRA-seventh-update-Outbreak-of-coronavirus-disease-COVID-19.pdf
COVID: coronavirus disease; ECDC: European Centre for Disease Prevention and Control; EU/EEA: European Union/European Economic Area.

